# Recurrent Jaundice Unraveled: A Case of Benign Recurrent Intrahepatic Cholestasis (BRIC) in an Indian Patient

**DOI:** 10.7759/cureus.74736

**Published:** 2024-11-29

**Authors:** Rachit Bansal, Abhay Patel, Chinmaya Singh, Kiran N C, Sonakshi Saxena

**Affiliations:** 1 General Medicine, Vardhman Mahavir Medical College and Safdarjung Hospital, New Delhi, IND; 2 General Medicine, Felix Hospital, Noida, IND

**Keywords:** benign recurrent intrahepatic cholestasis, bric, cholestasis, jaundice, pruritus, recurrent jaundice, ursodeoxycholic acid

## Abstract

Benign recurrent intrahepatic cholestasis (BRIC) is a rare, autosomal recessive liver disorder characterized by intermittent episodes of cholestasis without progression to chronic liver disease or cirrhosis. Patients experience recurrent jaundice and severe pruritus, significantly impacting their quality of life. This case report presents a 15-year-old boy with a history of recurrent jaundice and pruritus. The patient was successfully managed with ursodeoxycholic acid (UDCA), leading to symptomatic relief and biochemical improvement. BRIC is a rare disorder and should be considered in the differential diagnosis of recurrent cholestasis in adolescents. Early recognition and appropriate treatment can improve patient outcomes and prevent unnecessary investigations.

## Introduction

Benign recurrent intrahepatic cholestasis (BRIC) is an inherent cholestatic liver disease characterized by intermittent episodes of jaundice and pruritus that resolve spontaneously. First described by Summerskill and Walshe in 1959, BRIC poses a diagnostic challenge due to its rarity and nonspecific symptoms that often mimic other hepatic conditions [1]. The disorder is inherited in an autosomal recessive pattern and is classified into two types based on genetic mutations: BRIC type 1, associated with mutations in the ATP8B1 gene, and BRIC type 2, linked to mutations in the ABCB11 gene [1].

BRIC episodes can last from weeks to months and are followed by asymptomatic periods that may extend for years. BRIC is usually considered benign because it typically does not progress to cirrhosis. Still, the recurrent nature and severity of symptoms can significantly impair the quality of life, and close follow-up is necessary due to its close resemblance to progressive familial intrahepatic cholestasis (PFIC). PFIC1 is a disease spectrum that connotes three different disease states; Bayler disease and BRIC1 are differentiated based on different ages of disease onset, progression, and course. ICP1 is due to transient cholestasis only during pregnancy. Early recognition and management are essential to mitigate symptoms and prevent unnecessary interventions. This case report presents a case of a 15-year-old boy who presented with cholestatic jaundice with a history of recurrent jaundice in the past.

## Case presentation

A 15-year-old boy presented with a history of yellowish discoloration of the skin and sclera for two weeks, accompanied by generalized pruritus that interfered with his daily activities and sleep. There was no history of abdominal pain, fever, weight loss, pale stools, dark urine, or exposure to drugs. The patient complained of pruritus, which was severe in intensity and present throughout the day. The patient also had a past history of similar symptoms of jaundice and pruritus at the ages of six and 10, each lasting approximately two months and resolved spontaneously without extensive medical evaluation or treatment.

On examination, the patient appeared alert and well-developed for his age, with stable vital signs. He had marked icterus and excoriation marks on his limbs and trunk due to scratching. On systemic examination, there was no hepatosplenomegaly, ascites, or peripheral stigmata of chronic liver disease, such as spider angiomas, palmar erythema, gynecomastia, or dilated veins. Neurological examination was unremarkable, and there were no KF rings on slit lamp examination.

Laboratory investigations (Table 1) revealed elevated total bilirubin levels of 29.6 mg/dL, with a direct (conjugated) bilirubin predominance of 25.4 mg/dL. Liver enzymes showed elevated alkaline phosphatase (ALP, 830 U/L) and mildly increased alanine aminotransferase (ALT) and aspartate aminotransferase (AST) levels (85 and 90 U/L, respectively), indicative of a cholestatic pattern. Gamma-glutamyl transferase (GGT) was within normal limits, which is characteristic of BRIC because, in BRIC, there is predominantly intrahepatic cholestasis without biliary duct involvement [2]. Complete blood count and coagulation profile were within normal ranges.

**Table 1:** Laboratory reports ALT, alanine transaminase; ALP, alkaline phosphatase; ANA, anti-nuclear antibodies; AMA, anti-mitochondrial antibodies; AST, aspartate transaminase; GGT, gamma-glutamyl transferase; HAV, hepatitis A virus; HBsAg, hepatitis B surface antigen; HCV, hepatitis C virus; HEV, hepatitis E virus; INR, international normalized ratio; TLC, total leucocyte count

Parameter	Patient value	Reference range
Hemoglobin (g/dL)	13.4	11.5-17.0
TLC (cells/mm^3^)	8200	4000-10,000
Platelet count (x10^3^/mm^3^)	320	150-500
Total bilirubin (mg/dL)	29.6	0.3-1.2
Direct bilirubin (mg/dL)	25.4	
ALT (U/L)	85	10-45
AST (U/L)	90	10-35
ALP (U/L)	830	40-128
Anti-HAV IgM	Negative	
Anti-HEV IgM	Negative	
HBsAg	Non-reactive	
Anti-HCV	Non-reactive	
HIV-I/II	Non-reactive	
Ceruloplasmin (mg/dL)	21	14-40
24-h urinary copper excretion (mcg/24 h)	36	<60
GGT (IU/L)	28	10-45
Serum bile acids ( IU/L)	690	<15
Prothrombin time (seconds)	11.7	9.5-12.5
aPTT (seconds)	33.4	24.5-32.5
INR	1.05	
Serum protein (g/dL)	7.8	6.4-8.3
ANA	Negative	
AMA	Negative	
S.ACE level (IU/L)	20	<40

**Figure 1:**
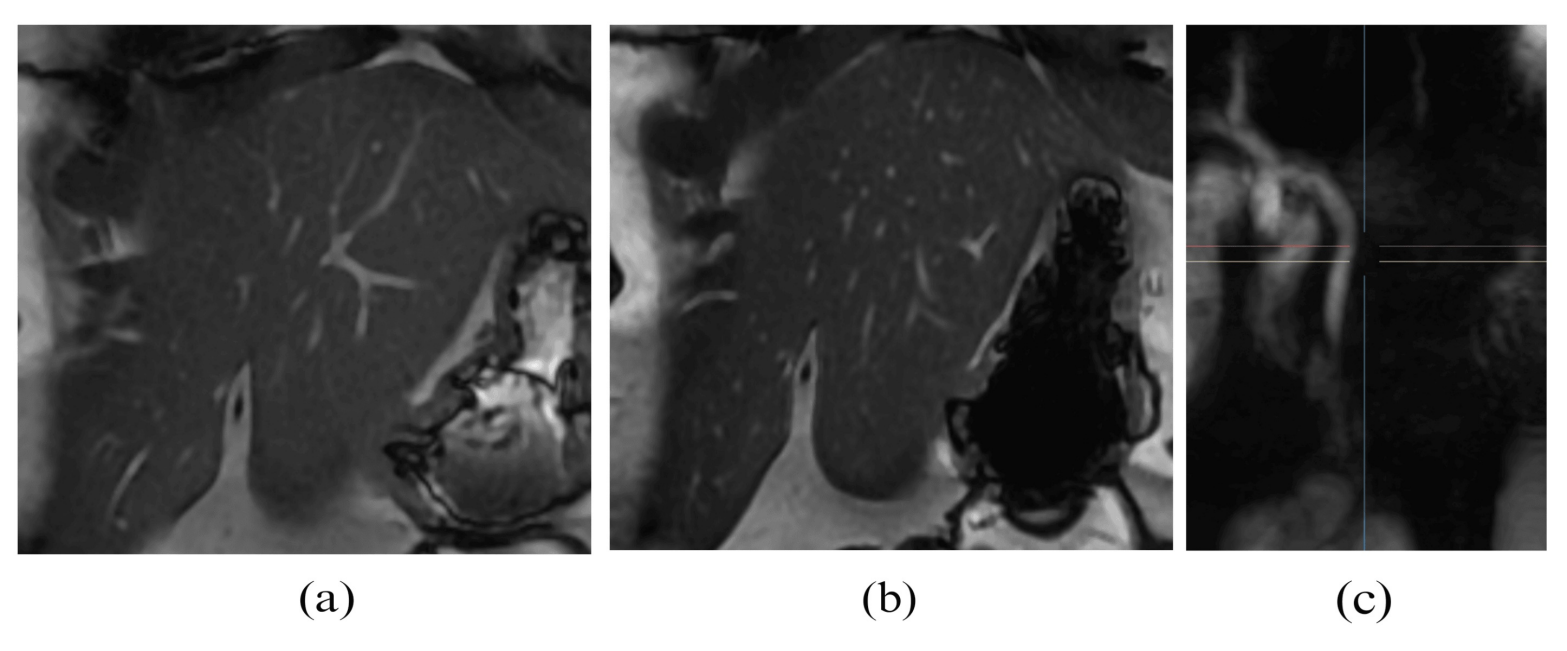
Magnetic resonance cholangiopancreatography revealing normal findings (a) Right and left hepatic ducts are normal. (b) No intrahepatic biliary dilatation. (c) Common bile duct is normal.

Serological tests (Table 1) for viral hepatitis A, B, C, and E were negative. Autoimmune markers, including antinuclear antibodies and anti-smooth muscle antibodies, were negative, reducing the likelihood of autoimmune hepatitis. Wilson’s disease was ruled out through normal serum ceruloplasmin levels and 24-hour urinary copper excretion. Abdominal ultrasound showed normal liver size and echotexture, with no evidence of biliary ductal dilatation or gallstones. Magnetic resonance cholangiopancreatography (MRCP) revealed normal intrahepatic and extrahepatic bile ducts without any obstructive pathology (Figure 1). After extensive evaluation, familial hyperbilirubinemia with predominantly conjugated hyperbilirubinemia was suspected.

**Figure 2:**
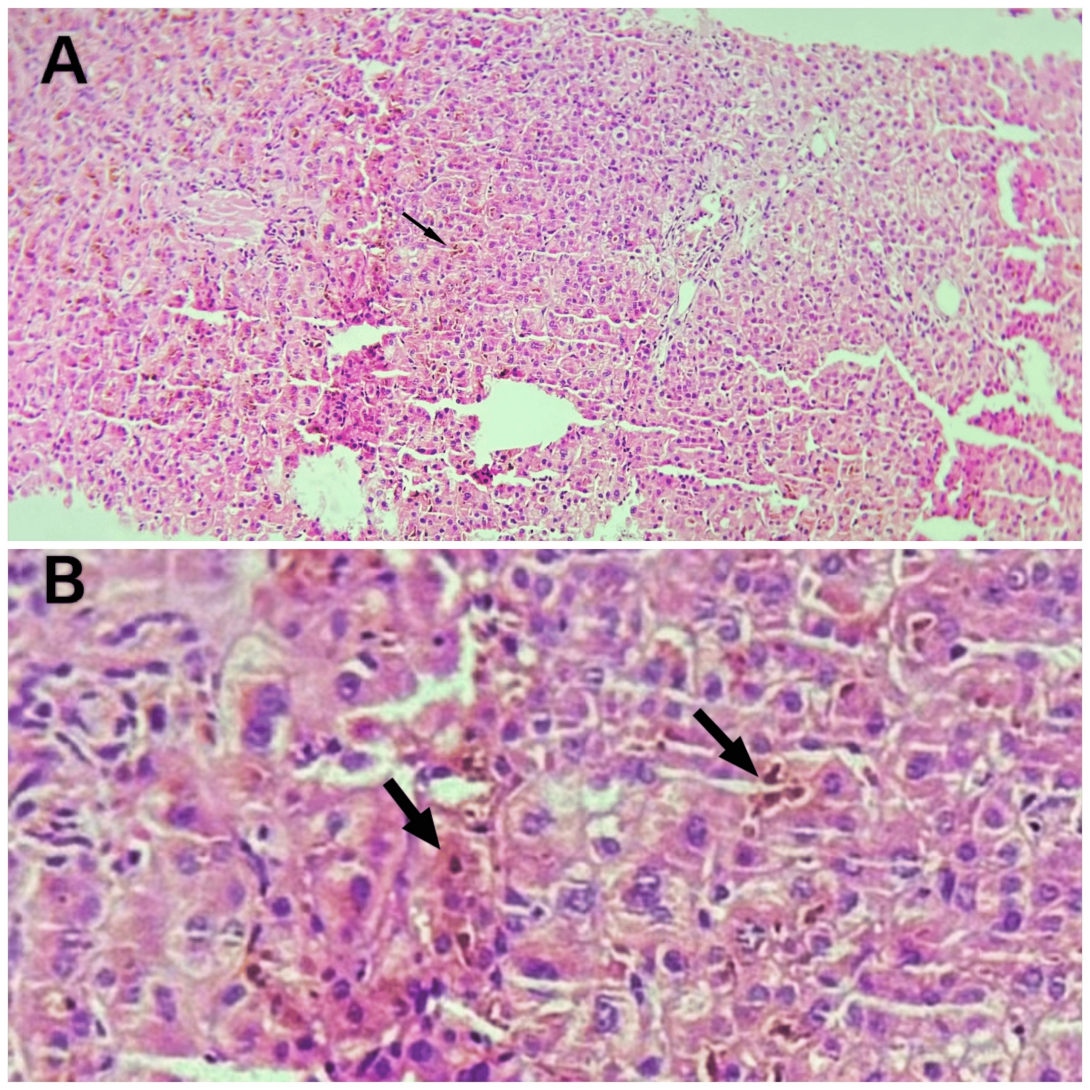
Liver biopsy showing intrahepatic cholestasis (arrow) on H&E staining (A,B) Sections examined from the liver core show liver parenchyma with evidence of intrahepatic cholestasis (arrow): no periportal inflammation or granuloma was identified on the biopsy sample.

Percutaneous liver biopsy was performed, suspecting intrahepatic cholestasis. Histopathological examination demonstrated intrahepatic cholestasis with bile plugs in the canaliculi, mild hepatocellular ballooning, and no significant inflammation or fibrosis. There were no features suggestive of autoimmune hepatitis, primary sclerosing cholangitis, or metabolic liver diseases. These findings were consistent with intrahepatic cholestasis without progressive liver damage (Figure 2).

Based on the recurrent episodes of cholestasis, exclusion of other causes of cholestasis, normal imaging studies, and histological findings, a diagnosis of BRIC was established. The Luketic and Shiffman diagnostic criteria for BRIC were fulfilled, supporting the diagnosis [3]. Although genetic testing for ATP8B1 and ABCB11 mutations would have provided definitive confirmation, it was not performed due to resource limitations.

The patient was managed conservatively with supportive care, including adequate hydration and nutritional support. Antihistamines were prescribed for pruritus but provided minimal relief. Given the severity of symptoms and impact on quality of life, ursodeoxycholic acid (UDCA) was initiated at 300 mg three times daily (approximately 15 mg/kg/day).

Within six days of starting UDCA therapy, the patient reported significant relief from pruritus. His sleep patterns improved, and he was able to resume normal activities. Serial liver function tests showed a gradual decline in total bilirubin levels from 29.6 mg/dL on admission to 20 mg/dL after two weeks and 4.6 mg/dL by the fourth week. The direct bilirubin levels and liver enzymes also showed corresponding decreases. After four weeks of hospitalization, the patient was discharged with instructions to continue UDCA therapy and attend regular outpatient follow-up appointments.

During the discharge counseling, the patient and his family were educated about BRIC’s benign but recurrent nature. They were informed about the possibility of future episodes and the importance of early medical consultation if symptoms reappeared. They were also explained about the low risk, but the potential progression to PFIC and the need for regular follow-up were discussed. Genetic counseling was offered, considering the autosomal recessive inheritance pattern, and the family was encouraged to seek evaluation for other siblings.

## Discussion

BRIC is a rare disorder with an estimated incidence of one in 50,000 to one in 100,000 individuals [3]. It is characterized by recurrent episodes of cholestasis that resolve spontaneously without leading to chronic liver disease or cirrhosis. The episodes are marked by jaundice, severe pruritus, fatigue, and malaise, significantly affecting patients’ quality of life.

The pathogenesis of BRIC involves genetic mutations that impair bile acid transport. BRIC type 1 is associated with mutations in the ATP8B1 gene, while BRIC type 2 is linked to mutations in the ABCB11 gene [1]. These genes encode proteins essential for normal bile formation and flow. Impaired function leads to the accumulation of bile acids within hepatocytes, causing cholestasis.

Diagnosis of BRIC requires a high index of suspicion. It is often a diagnosis of exclusion after ruling out other causes of cholestasis, such as viral hepatitis, autoimmune hepatitis, biliary obstruction, and metabolic liver diseases. Imaging studies are typically normal; liver biopsy shows intrahepatic cholestasis without significant inflammation or fibrosis.

Management is primarily supportive. UDCA is indicated for the management of cholestasis, although its efficacy varies among patients [1]. In this case, UDCA led to significant symptomatic and biochemical improvement. Other treatments for pruritus include cholestyramine, rifampicin, and, in refractory cases, plasmapheresis or molecular adsorbent recirculating system (MARS) therapy [4].

While BRIC is considered benign, there is a potential risk of progression to PFIC, a more severe condition leading to chronic liver disease and cirrhosis [5]. Long-term follow-up is essential to monitor for signs of disease progression. This case highlights the importance of considering BRIC in patients presenting with recurrent cholestasis. Early recognition and appropriate management can improve patient outcomes and prevent unnecessary diagnostic procedures or interventions.

## Conclusions

In conclusion, this case of a 15-year-old boy with BRIC emphasizes the need to consider this rare disorder in adolescents with recurrent jaundice and pruritus. The successful management of UDCA demonstrates the effectiveness of early intervention in alleviating symptoms and improving quality of life.

Increased knowledge and subsequent early understanding of BRIC can allow it to be differentiated from other liver diseases in the future and, therefore, ensure proper management and decrease unnecessary investigations.
